# Temozolomide-perillyl alcohol conjugate induced reactive oxygen species accumulation contributes to its cytotoxicity against non-small cell lung cancer

**DOI:** 10.1038/srep22762

**Published:** 2016-03-07

**Authors:** Xingguo Song, Li Xie, Xingwu Wang, Qian Zeng, Thomas C. Chen, Weijun Wang, Xianrang Song

**Affiliations:** 1 Shandong Provincial Key Laboratory of Radiation Oncology, Shandong Cancer Hospital and Institute, Jinan, Shandong, PR China; 2 Clinical Laboratory, Shandong Cancer Hospital and Institute, Jinan, Shandong, PR China; 3 School of Medicine and Life Sciences, University of Jinan, Shandong Academy of Medicine Science, Jinan, Shandong, PR China; 4 Departments of Neurological Surgery, and Pathology, University of Southern California, Los Angeles, California.

## Abstract

Temozolomide-perillyl alcohol conjugate (TMZ − POH), a novel temozolomide analog, was reported to play a cytotoxic role in triple-negative breast cancer and TMZ-resistant gliomas. In a current study we had demonstrated how TMZ − POH also exhibited its cytotoxicity against non-small cell lung cancer (NSCLC), the most common type of lung cancer, as evidence from cell/tumor proliferation inhibition, G2/M arrest, DNA damage and mitochondrial apoptosis. Importantly, TMZ − POH’s cytotoxicity is closely related to reactive oxygen species (ROS) accumulation because it can be reversed by two ROS scavengers, catalase (CAT) and N-acetyl-L-cysteine (NAC). TMZ − POH induces mitochondrial transmembrane potential (MTP) decrease and ROS accumulation, in turn activates mitogen-activated protein kinase (MAPKs) signaling and mitochondrial apoptosis, and then exerts its cytotoxicity, thus proposing TMZ − POH as a potential therapeutic candidate for NSCLC.

Non-small cell lung cancer (NSCLC), the most common type of lung cancer which accounts for about 80% of all lung cancer, has been shown to be a significant rising trend in terms of morbidity and mortality worldwide in recent years^1^. Despite of the continuous improvements in surgical resection, irradiation, chemotherapy and targeted therapy, NSCLC patients remain extremely vulnerable to relapse and mortality, and the overall 5-year survival rate still remains at 10 ~ 15%^2^. Chemotherapy has been traditionally considered as one of the standard treatment options for NSCLC, but the toxic side-effects and chemo-resistance hinder its clinical application.

Temozolomide (TMZ), an imidazotetrazine derivative of the alkylating agent dacarbazine, was first used to treat primary brain tumors^3^, such as glioblastoma multiforme and oligodendroglioma. Its antitumor activities were mainly due to its capability to react with DNA to form methyl adducts^4^, such as O^6^-methyl-guanine (O^6^-meG), which trigger cell cycle dependent DNA damage and cell death^5^, but these methyl adducts can be removed by O^6^-methylguanin-DNA-methltransferase (MGMT), ensuing TMZ resistance^6^. TMZ alone or combined with other agents was also tested as second (and more)-line therapy of NSCLC patients^7^,^8^, particularly in those with brain metastases^9^, although the potential benefits couldn’t be confirmed. For example, TMZ plus cisplatin followed by whole brain radiotherapy did not give better results than those usually achieved with other regimens^10^. Nevertheless, several advantages of TMZ over other conventional chemotherapeutic agents still attract the scientists’ attentions. It can cross the blood–brain barrier effectively due to its lipotropy^11^, and has less toxicity to the hematopoietic progenitor cells than other alkylating agents for its failure in causing chemical cross-linking of the DNA strands^6^. Therefore, it is a promising approach to develop a novel TMZ-derived chemotherapeutic agent against NSCLC.

Recent studies have demonstrated that perillyl alcohol (POH), a naturally occurring monoterpene which exerts promising antitumor activity in a variety of cancers including breast^12^, pancreas^13^, and lung carcinomas^14^, has the amazing capability to enhance the cytotoxicity of TMZ in several tumors including TMZ-resistant gliomas^15^. In this study, POH was covalently conjugated to TMZ, thus generating a novel TMZ analog, temozolomide-perillyl alcohol conjugate (TMZ − POH) (an honorable product from NeOnc Technologies). Previous researchers have already revealed that TMZ − POH displayed stronger anticancer potency than its individual constituents to several types of malignant neoplasms such as triple-negative breast cancer^16^ and TMZ-resistant gliomas^17^. However, the role and underlying mechanisms of TMZ − POH in NSCLC remain thoroughly unclear.

In the current study, we have demonstrated that TMZ − POH also exhibits anti-tumor activities against NSCLC more strongly than its individual constituents or their combination *in vitro* and *in vivo*. Importantly, the beneficial effects of TMZ − POH depend on it-induced reactive oxygen species (ROS) accumulation. ROS, the crucial modulators of cellular function^18^, is believed to play an important but complex role in cancer transformation and therapy^19^. A moderate ROS level can promote cell proliferation and differentiation^20^,^21^, whereas excessive amounts of ROS can lead to intracellular oxidative stress^22^. As excessive levels of ROS stress can also be toxic to the cells, cancer cells with increased oxidative stress are likely to be more vulnerable to damage by further ROS insults induced by exogenous agents^23^. Therefore, manipulating ROS levels by redox modulation is a way to selectively kill cancer cells without causing significant toxicity to normal cells^24^.

The overall hypothesis of the present study is that TMZ − POH-induced ROS is the key contributor to its anti-tumor activities, which triggers cell cycle-dependent DNA damage and G2/M arrest as well as downstream signal activation, and finally cell death, thus proposing TMZ − POH as a potential therapeutic candidate for NSCLC.

## Results

### TMZ − POH exerts stronger cytotoxicity than its individual constituents *in vitro* and *in vivo*

**Figure 1:**
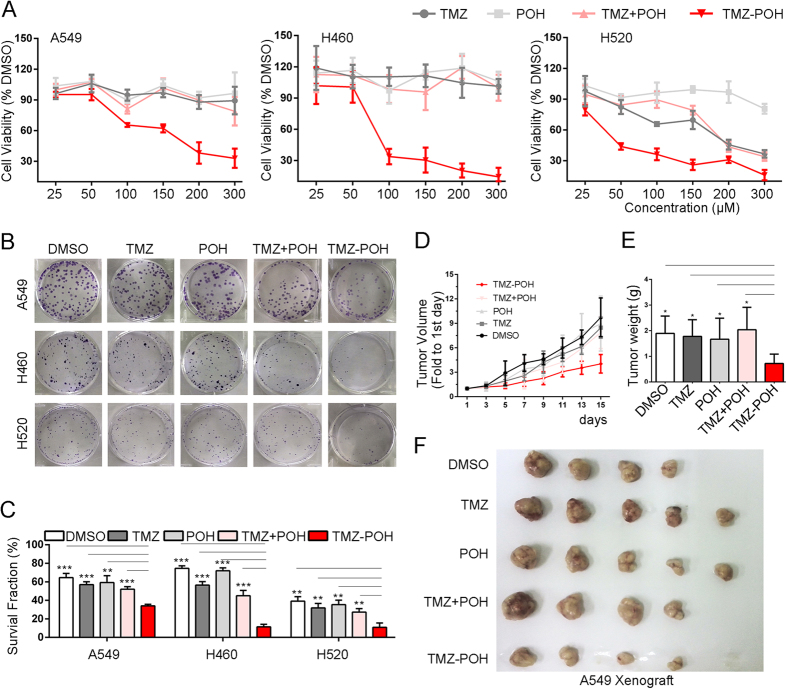
TMZ − POH exerts more cytotoxicity than its individual constituents *in vitro* and *in vivo*. (**A**) A549, H460 and H520 cells were treated with 0, 25, 50, 100, 150, 200, 300 μM TMZ, POH, TMZ + POH and TMZ − POH respectively for 48 h, DMSO acted as the control, and then subjected to MTT assay. Absorbance value was calculated and standardized to DMSO group. Three independent experiments were performed. (**B**) The above cells were treated with 100 μM TMZ, POH, TMZ + POH and TMZ − POH respectively for 14 days, DMSO acted as the control, and subjected to cell colony formation assay. (**C**) Statistical differences of cell colony formation assay were calculated and presented as mean ± SD. Three independent experiments were performed. (**D–F**) A549 xenograft tumor was established and treated as follows: DMSO, TMZ (25 mg/kg), POH (25 mg/kg), POH (11mg/kg) mixed with TMZ (14 mg/kg) (mimicking the dosage of the individual components contained in 25 mg/kg TMZ − POH), TMZ − POH (25 mg/kg). Tumor growth curves (**D**), tumor weight (**E**) and tumor image (**F**) of treated tumors with different treatment were detected. The results shown are means ± SD; *p < 0.05; **p < 0.01; ***p < 0.001.

To verify the cytotoxic potency of TMZ − POH against non-small cell lung cancer (NSCLC), three NSCLC cell lines A549, H460 and H520 were employed and exposed to treatment with control (Dimethyl Sulphoxide, DMSO), TMZ, POH, TMZ plus POH combination (TMZ + POH) and TMZ − POH, respectively. Cell viability detected by MMT assay was shown in Fig. 1A. TMZ − POH inhibited cell viability in a dose-dependent manner compared to its individual constituents and their combination in all three cancer cell lines, indicating the inhibitory effect of TMZ − POH on tumor cell proliferation independent of cell type. For H520 cells TMZ and TMZ + POH also exhibited a degree of growth suppressive effect, but more modest than TMZ − POH. In addition, colony formation was also suppressed by TMZ − POH more potently than its constituents and their combination. (Fig. 1B,C). These data support the inhibitory role of TMZ − POH in NSCLC cell growth and colony formation.

The inhibitory effect of TMZ − POH on the inhibition of NSCLC proliferation *in vivo* was also tested. Murine xenograft tumors were established and treated as described in ***“Materials and Methods”***. As expected, tumors administrated with TMZ − POH grew slower than those with other chemicals (Fig. 1D).The weight and volume of separated tumors were also smaller in TMZ − POH group than those in other drug-treated groups after the treatment completion (Fig. 1E,F), indicating that the therapeutic efficacy of TMZ − POH was substantially stronger than that of TMZ, POH, or their combination. No statistically significant differences in the body weights were observed between the mice in TMZ − POH-treated group and mice in control group (data not shown), indicating a low general toxicity of TMZ − POH. Taken together, the present study provides further insights into the cytotoxic effect of TMZ − POH against NSCLC cells *in vitro* and *in vivo*.

### TMZ − POH induces DNA damage and G2/M arrest

**Figure 2:**
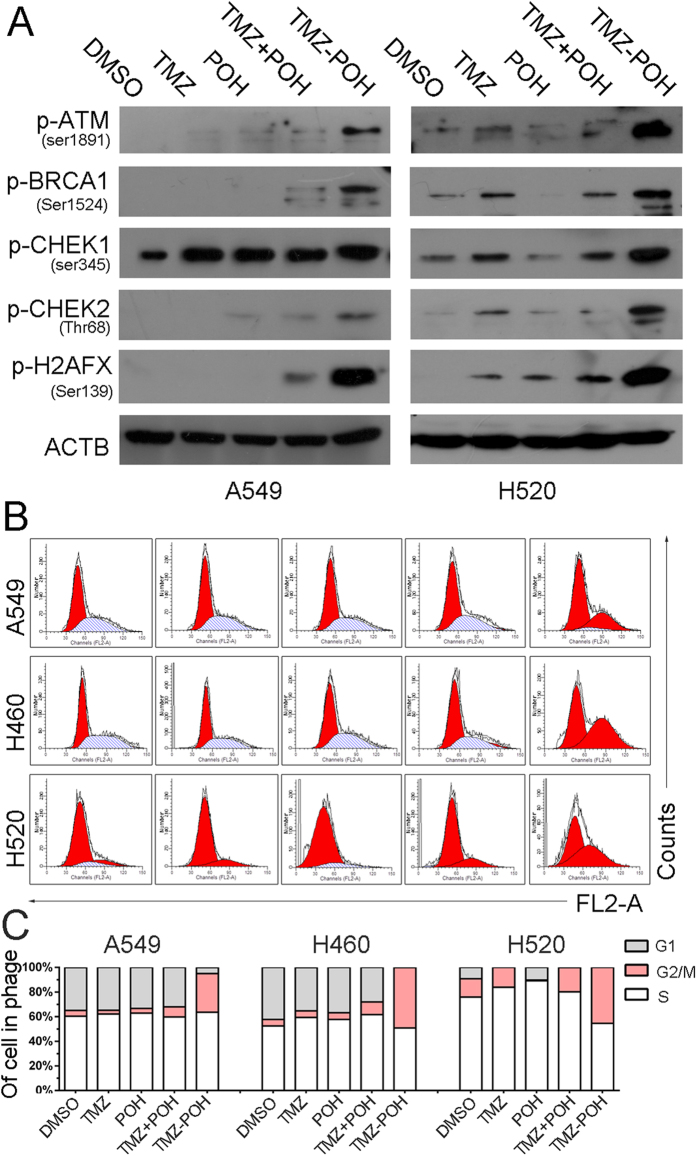
TMZ − POH induces DNA damage and G2M arrest. Cells were treated with 100 μM TMZ, POH, TMZ + POH, TMZ − POH or DMSO respectively for 48 h. (**A**) Western blot analysis demonstrated p-ATM, p-BRCA1, p-CHEK1, p-CHEK2, p-H2AFX and ACTB expression in above drug-treated A549 and H520 cells. (**B,C**) The cell cycle distributions of A549, H460 and H520 cells treated with above drugs were analyzed. Three independent experiments were performed.

One of underlying mechanisms of TMZ depends on its ability to alkylate DNA, which leads to DNA double strand breaks (DSBs) and DNA damage response (DDR)^25^. Therefore, whether TMZ − POH also triggered DNA damage-repair pathway was investigated. Ataxia telangiectasia mutated (ATM)^26^, a serine/threonine protein kinase, is activated following DNA damage and then transmits the DNA damage signal to downstream substrates, such as checkpoint kinases 1 and 2 (CHEK1/CHEK2)^27^. Indeed, TMZ − POH was observed to induce the phosphorylation of ATM at the Ser1981 site. CHEK1 and CHEK2, the cell cycle checkpoint kinases, were phosphorylated at the Ser345 and Thr68 site, respectively (Fig. 2A). Breast cancer 1 (BRCA1), an adaptor between ATM and CHEK1/CHEK2 to establish the cascade towards a checkpoint in the cell cycle, was phosphorylated at 1524 site^28^. When DNA is damaged, DSBs trigger the recruitment of ATM to the damaged site, which in turn phosphorylates H2A histone family member X (H2AFX) resulting in foci formation at the damage side^29^. As shown in Fig. 2A, TMZ − POH treatment also induced an increase in phosphorylated H2AFX (Ser139).

It has been reported that TMZ causes cell cycle arrest at G2/M through activation of ATM-CHEK1/2 signaling^30^. The above results also demonstrated TMZ − POH induced an increase in the amount of CHEK1, CHEK2 and BRCA1 (Fig. 2A), indicating the effect of TMZ − POH on cell cycle. To confirm this issue, cell cycle analysis was applied. As shown in Fig. 2B,C, TMZ − POH increased the population at the G2/M phase and decreased the population at the G1 in all tested NSCLC cells. As predicted from the results from cell viability assay, TMZ and TMZ + POH also induced a modest arrest at G2/M in H520 cells. Taken together, these results characterize TMZ − POH as an alkylating agent similar to TMZ, but with a stronger potency than the original compounds.

### TMZ − POH induces cell death via mitochondrial pathway

**Figure 3:**
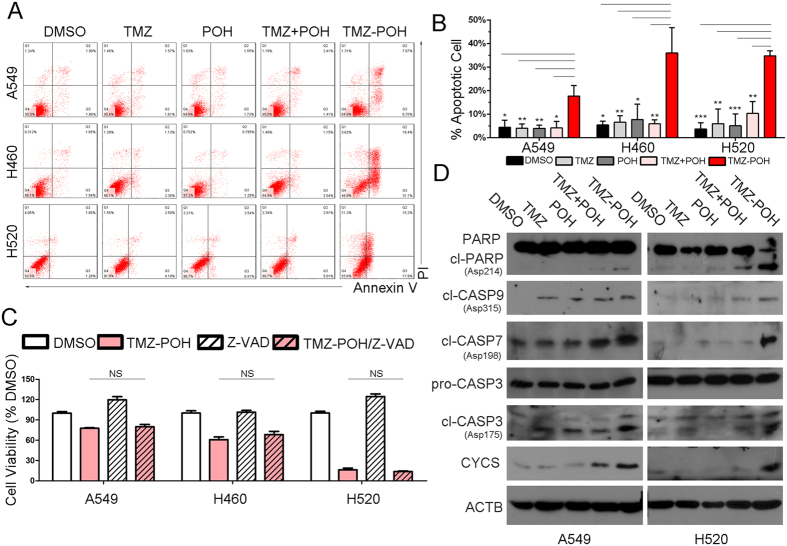
TMZ − POH induces cell death via mitochondrial pathway. (**A**) A549, H460 and H520 cells were treated with 100 μM TMZ, POH, TMZ + POH, TMZ − POH or DMSO respectively for 48 h and subjected to apoptosis assay using Annexin-V &PI staining. Three independent experiments were performed and the results statistically analyzed as means ± SD (**B**). (**C**) Above cells were treated with 100 μM TMZ − POH for 48 h with or without presence of z-Vad-Fmk (Z-VAD), and cell viability was detected by MTT assay. Two independent experiments were performed. (**D**) Lysates from drug-treated A549 and H520 cells (DMSO, TMZ, POH, TMZ + POH and TMZ − POH) were subjected to western blot using following antibodies against PARP (full and cleaved PARP), cleaved CASP9, cleaved CASP7, pro-CASP3, cleaved CASP3, Cytochrome C (CYCS) and ACTB. cl = cleaved; ns = no significance. The results shown are means ± SD; ***p < 0.001.

To determine whether TMZ − POH-treated tumor cells underwent cell death, apoptosis detection assay was performed. As shown in Fig. 3A,B, flow cytometry analysis displayed much more apoptotic or dead cells when treated with TMZ − POH other than treated with its individual constituents and their combination. To confirm the forms of TMZ − POH-induced cell death, Z-VAD-FMK (20 μM, Z-VAD), a pan-caspase inhibitor, was introduced in cell growth analysis. Unexpectedly, Z-VAD failed to prevent cells from TMZ − POH cytotoxicity (Fig. 3C), indicating that TMZ − POH might induce cell death in a caspase-independent manner.

Furthermore, apoptosis-related gene expression was detected in A549 and H520 cells. As shown in Fig. 3D, TMZ − POH treatment led to an increase in expression of cleaved caspase 3, 7 (CASP 3/7) and Poly (ADP-ribose) polymerases (PARP) but no influence on pro-caspase 3 (pro-CASP 3). Importantly, Cytochrome C (CYCS) and cleaved caspase 9 (cl-CASP 9), vital markers in mitochondrial pathways^31^, were also elevated significantly in response to TMZ − POH treatment (Fig. 3D), indicating that mitochondrial apoptosis is activated and plays a prominent role in TMZ − POH-mediated cell death. Therefore, the results from the apoptosis assay (Fig. 3) were correlated closely with those from cell viability, DNA damage and cell cycle arrest (Figs 1 and 2), and in all cases TMZ − POH clearly exhibited the strongest anti-cancer capability.

### TMZ − POH induces mitochondrial transmembrane potential (Δψm) decrease and ROS accumulation

**Figure 4:**
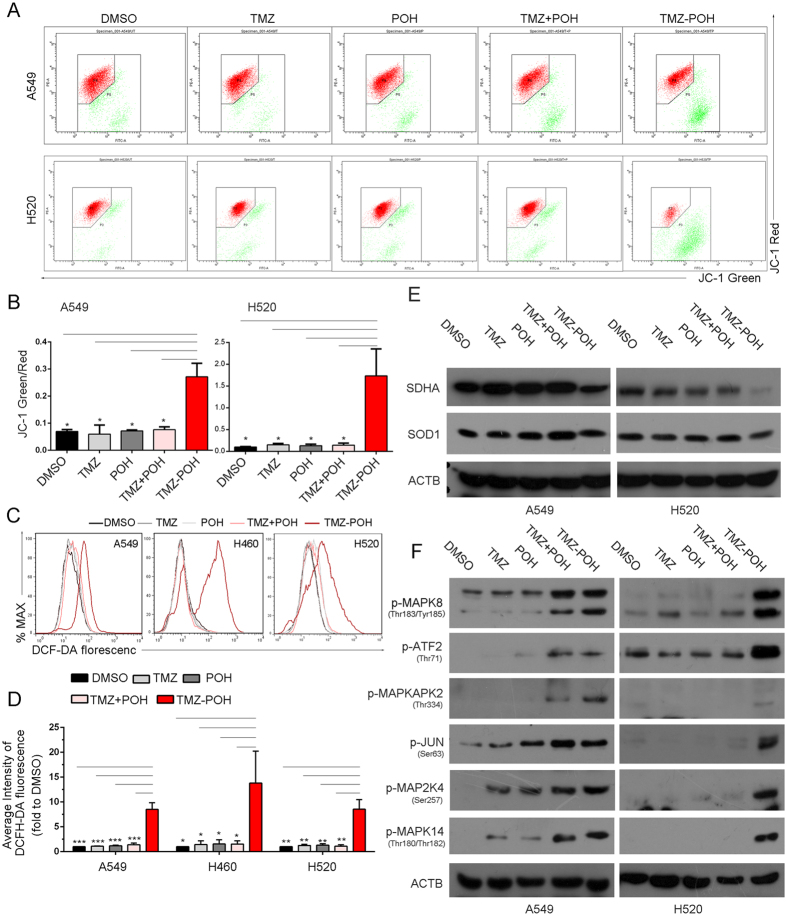
TMZ − POH induces mitochondrial transmembrane potential (Δψm) decrease and ROS accumulation. (**A,B**) A549 and H520 cells were treated with 100 μM TMZ, POH, TMZ + POH, TMZ − POH or DMSO for 48 h respectively, and detected using JC-1 flow cytometry (**A**) and the results statistically analyzed (**B**). Two independent experiments were performed. (**C,D**) Intracellular ROS levels were measured using the fluorescent probe DCFH-DA after cells were treated with the indicated constituents (100 μM) for 24 hours in A549, H460 and H520 cells (**C**). Three independent experiments were performed and the results statistically analyzed (**D**). (**E,F**) Lysates from above drug-treated A549 and H520 cells were subjected to western blot to detect intracellular antioxidant systems including SDHA, SOD1 and ACTB (**E**) and MAPKs signaling proteins including p-MAPK8, p-ATF2, p-MAPKAPK2, p-JUN, p-MAP2K4, p-MAPK14 and ACTB (**F**). The results shown are means ± SD; *p < 0.05; **p < 0.01; ***p < 0.001.

It is well established that mitochondria are important participants in the regulation of cell death^32^, the collapsed mitochondrial transmembrane potential (Δψm) also acts as a marker of apoptosis^33^. Next Δψm was evaluated by JC-1 probe (5,5′,6,6′-Tetrachloro-1,1′,3,3-tetraethylimidacarbocyanine iodide) in our study. As shown in Fig. 4A, TMZ − POH led to a significant increase in population of monomer JC-1 compared to other drugs, as evidence from an increase of JC–1 fluorescence signal ratio (the fluorescent intensity of green to red, Fig. 4B), indicating a collapse in mitochondrial membrane potential induced by TMZ − POH.

The mitochondrion is a major site of ROS generation in mammalian^18^, thus intracellular ROS levels were also measured using the 2′,7′-dichlorofluorescein diacetate (DCFH-DA). As shown in Fig. 4C,D, ROS levels increased significantly when treated with TMZ − POH but not with other drugs in A549, H460 and H520 cells, indicating a universal phenomenon that TMZ − POH induced ROS accumulation independent of cell type. Consistently, some components of intracellular antioxidant systems such as superoxide dismutase 1 (SOD1) and succinate dehydrogenase complex subunit A (SDHA) were also detected by western blot. Their expression wassignificantly suppressed by TMZ − POH other than other drugs (Fig. 4E). Recent researches have shown that mitogen-activated protein kinase (MAPKs) signaling plays a crucial role in response to ROS accumulation^34^, hence MAPKs signaling pathway was detected to further confirm TMZ − POH induced ROS accumulation. As shown in Fig. 4F, TMZ − POH increased phosphorylation of mitogen-activated protein kinase 8 (MAPK8, also called JNK), activating transcription factor 2 (ATF2), mitogen-activated protein kinase-activated protein kinase 2 (MAPKAPK2), jun proto-oncogene (JUN, also called c-Jun), mitogen-activated protein kinase kinase 4 (MAP2K4, also called MKK4), mitogen-activated protein kinase 14 (MAPK14, also called P38), indicating MAPKs were also activated by TMZ − POH. Collectively, our data suggest that TMZ − POH leads to an imbalance of the cellular redox potential and ultimately mitochondrial dysfunction.

### TMZ − POH induced cytotoxicity is blocked by CAT and NAC

**Figure 5:**
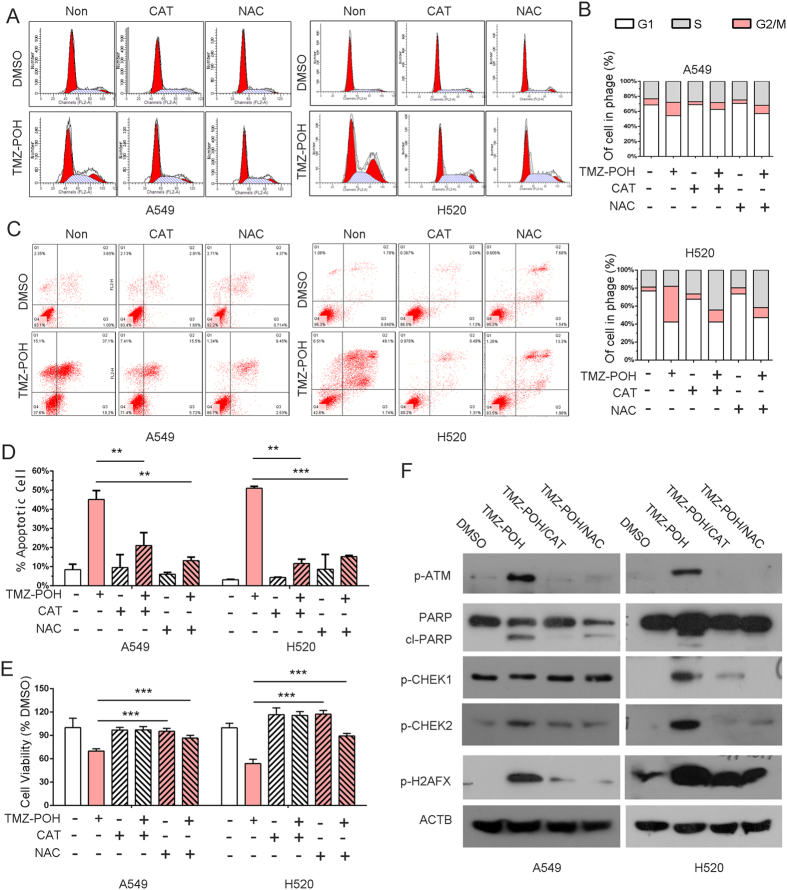
TMZ − POH induced cytotoxicity is blocked by CAT and NAC. (**A–E**) A549 and H520 cells were treated with 100 μM TMZ − POH for 48 h with or without presence of catalase (CAT) and N-acetyl-L-cysteine (NAC), and then cell cycle progression (**A,B**), apoptosis assay using Annexin-V &PI staining (**C,D**) and cell viability using MTT assay (**E**) were detected. Three independent experiments were performed. (**F**) Lysates from above drug-treated cells (DMSO, TMZ − POH) were subjected to western blot using following antibodies against p-ATM, PARP, p-CHEK1, p-CHEK2, p-H2AFX and ACTB. The results shown are means ± SD; ***p < 0.001.

To certify the contribution of TMZ − POH induced ROS accumulation to its cytotoxicity, we pre-treated A549 and H520 cells with two ROS scavengers, catalase (CAT, 1U) and N-acetyl-L-cysteine (NAC, 5 nM). After efficiently blocking-up of ROS accumulation, TMZ − POH’s cytotoxicity was significantly alleviated. As shown in Fig. 5A–E, G2/M arrest (Fig. 5A,B), apoptosis (Fig. 5C,D) and cell proliferation inhibition (Fig. 5E) observed upon treatment with TMZ − POH were significantly restored in the presence of CAT or NAC in both A549 and H520 cells. Taken together, the ability of ROS scavengers to abolish TMZ − POH-induced cytotoxicity suggests that ROS accumulation mainly contributes to its cytotoxicity against NSCLC.

Accumulating evidences support that ROS is involved in mitochondrial apoptosis and DNA damage response^35^. Therefore, we detected the related protein expression in the presence of CAT and NAC. As shown in Fig. 5F, TMZ − POH failed to increase expression of PARP, pho-ATM, pho-H2AFX, pho-CHEK1 and pho-CHEK2 when cells were co-treated with CAT or NAC, implicating ROS accumulation is the key contributor to TMZ − POH’s cytotoxicity.

## Discussion

In this study, we have demonstrated the cytotoxic effect of TMZ − POH on NSCLC. TMZ seemed not to provide conspicuous therapeutic effect for NSCLC patients despite of administration in second (or more) line therapy, whereas the newly designed compound TMZ − POH was markedly more cytotoxic than its constituents and their combination against NSCLC, as evidence from stronger inhibition of cell/tumor proliferation and colony formation, along with higher level of G2/M arrest, apoptosis and DNA damage, thereby proposing TMZ − POH as a potential candidate for NSCLC therapy.

Interestingly, our data showed TMZ and TMZ + POH also seemed to play a modest role in G2/M arrest, apoptosis and DNA damage in H520 but not in A549 cells. The underlying basis for these inconsistent results was not investigated, but it was conceivable that these differences might result from variable expression levels of O^6^-methylguanine-DNA methyltransferase (MGMT), A549 expressed high level of MGMT whereas H520 expressed low (Data not shown). TMZ’s cytotoxicity in A549 cells could be suppressed by MGMT via removal of O^6^-meG^6^, a DNA repair enzyme that has been demonstrated to contribute to treatment resistance. Contrast to TMZ and TMZ + POH, TMZ − POH exerts its cytotoxicity independent of the MGMT expression status, thus indicating a potential role of TMZ − POH in TMZ-resistant cells.

The superior activity of TMZ − POH attributes to its unique mechanisms. Although redox alterations in cancer cells are very complex and ROS-generating agents may not always lead to killing cancer cells^24^, several lines of evidence still validate TMZ − POH-induced ROS accumulation is the key contributor to its cytotoxicity. Firstly, TMZ − POH induced ROS accumulation in all tested cell lines, indicating a universal phenomenon independent of the cell types. Correspondingly, some components of intracellular antioxidant systems such as SOD1 and SDHA were significantly suppressed by TMZ − POH compared to other drugs. Secondly, mitochondria are the major source of ROS generation^18^, the behavior and function of mitochondria as well as mitochondrial apoptosis is closely dependent upon mitochondrial ROS (mtROS)^36^. Coincidently, TMZ − POH led to mitochondrial membrane potential collapse, along with activated mitochondrial apoptosis pathway, suggesting mitochondria might be the primary site TMZ − POH worked on, which induced mitochondrial membrane potential collapse, and in turn ROS accumulation and mitochondrial apoptosis. Thirdly, TMZ − POH could activate MAPKs signaling, which is reported to be activated signal when ROS accumulated. Finally, TMZ − POH-induced cytotoxicity was efficiently prevented by two ROS scavengers, CAT and NAC, as evidence from restoration of cell viability, G2/M arrest, apoptosis and DNA damage. Notably, DNA damage induced by TMZ − POH slightly differed from that by TMZ. As an alkylating agent dacarbazine, TMZ works due to its capability to methylate DNA directly^37^, whereas our data supported DNA damage induced by TMZ − POH resulted from ROS accumulation, because it could be repaired when ROS scavenged. However, whether TMZ − POH also methylates DNA like an alkylating agent needs further investigation. Taken together, the current study supports TMZ − POH exhibits cytotoxicity via ROS accumulation.

Collectively, we add a novel insight into the role and mechanisms of TMZ − POH in NSCLC, a newly designed compound based on TMZ conjugated with POH. We have demonstrated that TMZ − POH exhibited its cytotoxicity against NSCLC via ROS accumulation, which resulted from mitochondrial membrane potential collapse and led to mitochondrial apoptosis, DNA damage, cell cycle arrest and inhibited tumor proliferation, thereby indicating a promising therapeutic agent against NSCLC.

## Materials and Methods

### Pharmacological agents

Temozolomide (TMZ) was purchased from Sigma-Aldrich (34219, Shanghai, China) and dissolved in DMSO (Sigma-Aldrich, D2650, Shanghai, China) to a concentration of 100 mM. Perillyl alcohol (POH) and TMZ − POH were provided by Neonc Technologies, Inc. (Los Angeles, USA) and diluted with DMSO to make stock solutions of 100 mM. In all cases of cell treatment, the final DMSO concentration in the culture medium never exceeded 0.5%. Stock solutions of all drugs were stored at −20 °C.

### Cell culture and Treatment

Human non-small cell lung cancer (NSCLC)-derived cell lines A549, H460 and H520 were purchased from American Type Culture Collection (Manassas, VA, USA) and China Center for Type Culture Collection (Wuhan, China). All these cells were grown in DMEM (Gibco/Invitrogen, C11995500CP, Carlsbad, CA, USA) supplemented with 10% fetal bovine serum (Gibco/Invitrogen, 10099-141) and antibiotics (penicillin/streptomycin, 100 U/ml, Gibco/Invitrogen, 15140-122) at 37 °C in 5% CO_2_. Cells were plated in cell culture plates and allowed to adhere overnight, subsequently treated by DMSO, TMZ, POH, TMZ + POH and TMZ − POH (all 100 μM) for 48 hours, respectively. In some experiments, ROS scavengers, catalase (CAT, Sigma-Aldrich, C1345) and N-acetyl-L-cysteine (NAC, Sigma-Aldrich, A7250) were employed 2 hours before the above treatments.

### MTT assay

Cells were plated in 96-well-plates at 3,000–4,000 per well (Corning, USA) dependent on cell type. After drug treatment for 48 h, MTT assay was done through the addition of 3-(4,5-dimethylthiazol-2-yl)-2,5-diphenyltetrazolium bromide) solution (made by adding 5 mg/mL in PBS) at 10 μl per well, followed by incubation at 37 °C in 5% CO_2_ for 4 hours. Formazan crystals that formed were solubilized with 100 μl of acidified (0.01 M HCl) 10% SDS (sodium dodecyl sulfate) overnight at 37 °C. Absorbance (A) at 570 nm (reference wavelength: 630 nm) was read on a Bio-Rad 680 microplate reader (Bio-rad 680, Bio-Rad Laboratories, Hercules, USA). The cell viability was calculated with the following formula: cell viability = A^drug-treated^/A^DMSO^ ×100%. In some experiments, Z-VAD (20 μM) was added to blocked caspase dependent apoptosis. The experiment was repeated three times.

### Colony formation assay

Depending on the cell line, 200–350 cells were seeded into each well of a 6-well plate (Corning, USA) and exposed to above drugs, and then incubated at 37 °C for 14 days. After fixation with acetic acid–methanol (1:4) and staining with diluted crystal violet (1:30), colonies that consisted of >50 cells were counted and calculated. The colony formation efficiency was calculated with the following formula: Survival Fraction = Clones/Cell numbers × 100%. Each cell was repeated in three wells and the experiment was repeated three times.

### Cell cycle analysis

Cells growing in 12-well plates were treated by above agents, cells were collected and washed once with PBS (137 mM NaCl, 2.7 mM KCl, 8 mM Na_2_HPO_4_, 2 mM KH_2_PO_4_, pH 7.4), and then re-suspended and fixed in 70% ethanol overnight. After incubation in 1 ml of propidium iodide staining solution (0.1% Triton X-100, 200 μg/ml DNase-free RNase A, 20 μg/ml propidium iodide) for 1 hour at room temperature, DNA content was evaluated by a FACS Calibur instrument (Becton Dickinson, Bedford, MA, USA) and the distribution of cell cycle phases were determined using ModiFit software (Topsham, ME, USA). Three independent experiments were carried out.

### Apoptosis analysis

Apoptosis was evaluated by using the Annexin V-FITC Apoptosis Detection Kit (BD Biosciences Pharmingen, 556547, San Diego, USA) according to the description provided by the manufacturer. 1.5 × 10^5^ cancer cells grown in six well plates overnight were exposed to above treatment at indicated concentrations for an additional 48 h. After drug treatment, the cells were trypsinized, collected and stained with FITC-Annexin V & Propidum Iodide (PI) for 15 minutes in the dark. The stained cell population were determined using by a FACS Calibur instrument (Becton Dickinson, USA) and the data were analyzed using FlowJo Software 7.6 (Treestar, Inc., San Carlos, CA). Three independent experiments were carried out.

### Determination of ROS accumulation

ROS accumulation in cells following the above treatment were evaluated using 2′, 7′-dichlorofluorescein diacetate (DCFH-DA) kit (Beyotime, S0033, Beijing, China) according to the manufacturer’s protocol. Briefly, after indicated treatment for 48 h, the cancer cells were washed with serum free DMEM twice and incubated with DCF-DA (20 μM, diluted in serum free DMED) for 20 minutes at 37 °C in 5% CO_2_, and then collected and suspended in PBS. The florescence intensity of dichlorofluorescein was measured by a FACS Calibur instrument (Becton Dickinson, USA) with the excitation source at 488 nm and emission at 525 nm and the data were analyzed using FlowJo Software 7.6 (Treestar, Inc., CA). Three independent experiments were carried out.

### Analysis of mitochondrial transmembrane potential (Δψm)

1.5 × 10^5^ cancer cells grown in six well plates overnight were exposed to above indicated, then stained with the cationic dye 5,5′, 6,6′-Tetrachloro-1,1′, 3,3′-tetraethyl-imidacarbocyanine iodide (JC-1; C2006, Beyotime, China) to demonstrate the state of mitochondrial transmembrane potential according to the manufacturer’s protocol. Briefly, cells were harvested and transferred to 1.5 ml tubes, and then incubated with JC-1 (5 μg/ml) in a 37 °C incubator for 20 minutes after washing twice with PBS. Subsequently, cells were collected and subjected to flow cytometry (Becton Dickinson, USA) to detect the change of JC-1 florescence. The data were analyzed using BD FACS DIVA software (Becton Dickinson, USA). The mitosensor dye JC-1 aggregates in the normal mitochondria, whereas JC-1 is not able to accumulate in the mitochondria and remains as monomers emitting green fluorescence in the collapsed mitochondria^33^. Two independent experiments were carried out.

### Western blots

Cells were lysed in Cell Lysis Buffer (20 mM Tris pH 7.5, 150 mM NaCl, 1% Triton X-100) (Beyotime, P0013, Beijing, China) supplemented with 0.5 mM phenylmethanesulfonyl fluoride (PMSF, Beyotime, ST506), and the total cellular protein concentration was determined with a BCA Protein Assay Kit (Thermo Fisher Scientific Inc., 23227, Rockford, USA). 50 μg quantity of protein was separated on SDS-PAGE and transferred onto PVDF membranes (Millipore, Billerica, MA, USA). Membranes were then blocked with 5% evaporated skimmed milk in Tris-buffered saline (50 mM TRIS-HCl, pH 7.5, 150 mM NaCl) containing 0.1% Tween-20 for 1 hour, and probed overnight at 4 °C with the following primary antibodies: antibodies against human phospho-ATM (5883), PARP (9532), phospho-BRCA1 (9009), phospho-H2AFX (9718), phospho-CHEK1/2 (2348/2661), CYCS (11940), MAPK8 (4668), ATF2 (5112), MAPKAPK2 (3007), JUN (3270), MAP2K4 (4514), MAPK14 (4511), SDHA (11998), SOD1 (4266), cleaved-CASP 3/7/9 (9664/8438/9505) (all 1:1000; Cell Signaling Technology, Danvers, MA, USA), antibody against ACTB (1:2000; Zsbio, sc-53142, Beijing, China), followed by incubation with horseradish peroxidase coupled secondary anti-mouse (Zsbio, ZB-2305) or anti-rabbit antibodies (Zsbio, ZB-2301) for 1 hour at room temperature. The protein bands were visualized using ECL blotting detection reagents (Beyotime, P0018), and developed and fixed onto x ray films. ACTB was served as a loading control.

### *In vivo* studies

BALB/c nude mice (4–6 weeks of age, female) were purchased from Beijing HFK Bioscience Co., Ltd. (Beijing, China). Mice were housed and handled in laminar flow cabinets under specific pathogen-free conditions according to institutional guidelines and experimental procedures approved by the Institutional Animal Care and Use Committee of Shandong Cancer Hospital and Institute with full respect to the EU Directive 2010/63/EU for animal experimentation. Approximately 5 × 10^6^ A549 cells in 100 μl PBS were innoculated s.c. into the left flank of nude mice. When reached approximately 100 mm^3^ in size 1 week later, mice were randomly divided into five groups, and treated once a day for 15 days as follows: DMSO, TMZ, POH, TMZ + POH and TMZ − POH. Size of local tumors was calculated by measuring two perpendicular diameters (length and width) every two days using a caliper, and the volume was calculated according to the formula: tumor volume (mm^3^) = 1/2 × (length × square width) as described previously^38^. The mice were sacrificed after completion of treatment and the tumors were separated and weighted.

### Statistical analysis

Statistical significance was evaluated with data from at least three independent experiments. GraphPad Prism 6.02 (GraphPad Software, San Diego, CA, USA) was used for data analysis. Statistical analysis was carried out using student *t*-test. Data are presented as the mean ± SD. For all statistical tests, significance was established at P < 0.05.

## Additional Information

**How to cite this article**: Song, XG. *et al.* Temozolomide-perillyl alcohol conjugate induced reactive oxygen species accumulation contributes to its cytotoxicity against non-small cell lung cancer. *Sci. Rep.* **6**, 22762; doi: 10.1038/srep22762 (2016).

## Supplementary Material

Supplementary Information
