# Supplementary material for: Temozolomide-perillyl alcohol conjugate induced reactive oxygen species accumulation contributes to its cytotoxicity against non-small cell lung cancer
Source: Sci Rep. 2016 Mar 7;6:22762. doi: 10.1038/srep22762 (PMC4780103; doi:10.1038/srep22762)
Supplement: Supplementary Information [file srep22762-s1.pdf]

Supplementary Information

Temozolomide-perillyl alcohol conjugate induced reactive oxygen species accumulation contributes to its cytotoxicity against non-small cell lung cancer

Song Xingguo [#]; Xie Li [#]; Wang Xingwu; Zeng Qian; Thomas C. Chen; Weijun Wang; Song Xianrang [*]

# These authors contributed equally to this work

* Correspondence should be addressed to Dr. Song Xianrang:

Shandong Provincial Key Laboratory of Radiation Oncology, Shandong Cancer Hospital and Institute, 440 Ji-Yan Road, Jinan 250117, Shandong Province, PR China; Email: basiclab@163.com; Tel: +86-531-62626421

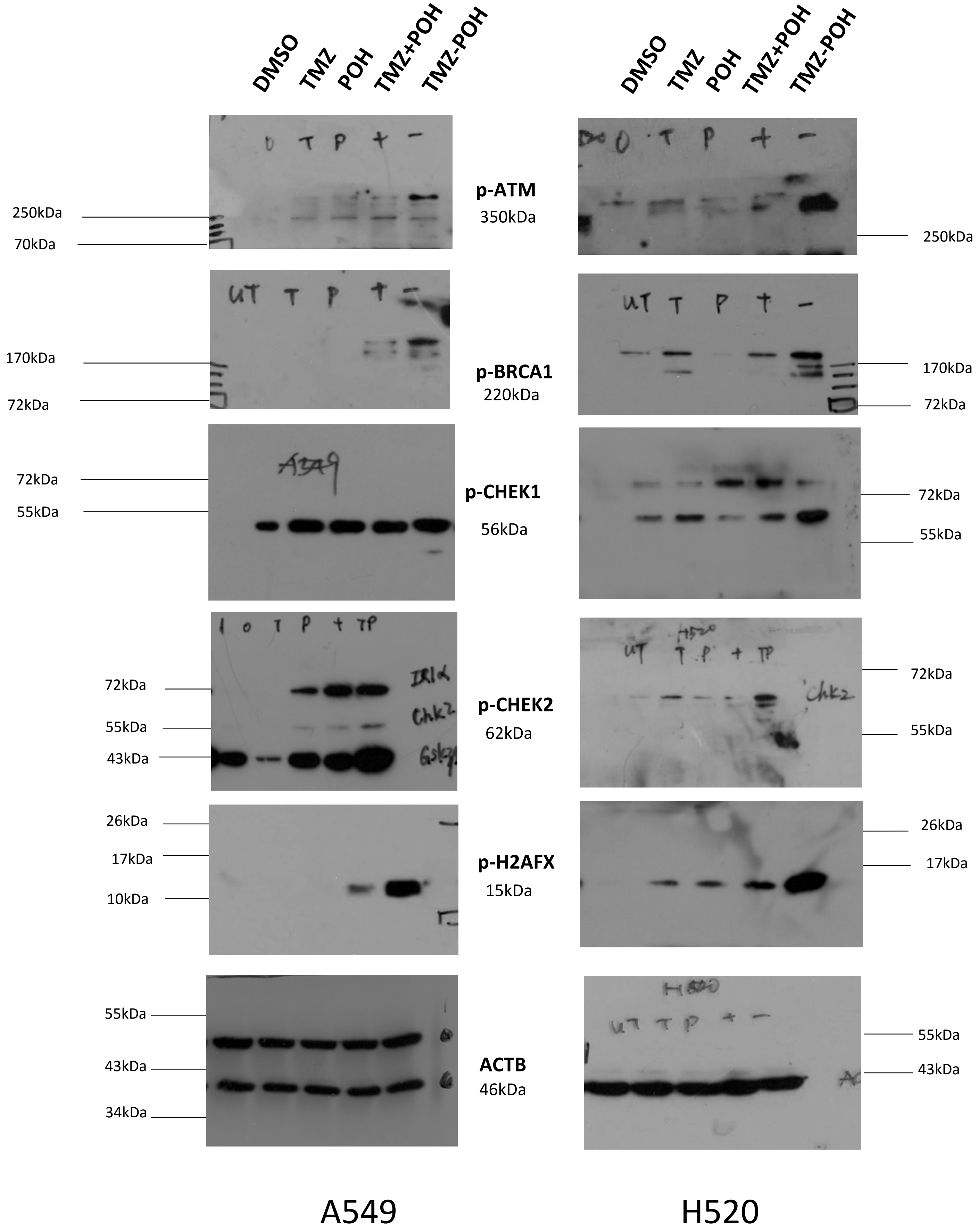

Supplement Figure S1

Supplementary File for Figure 2A

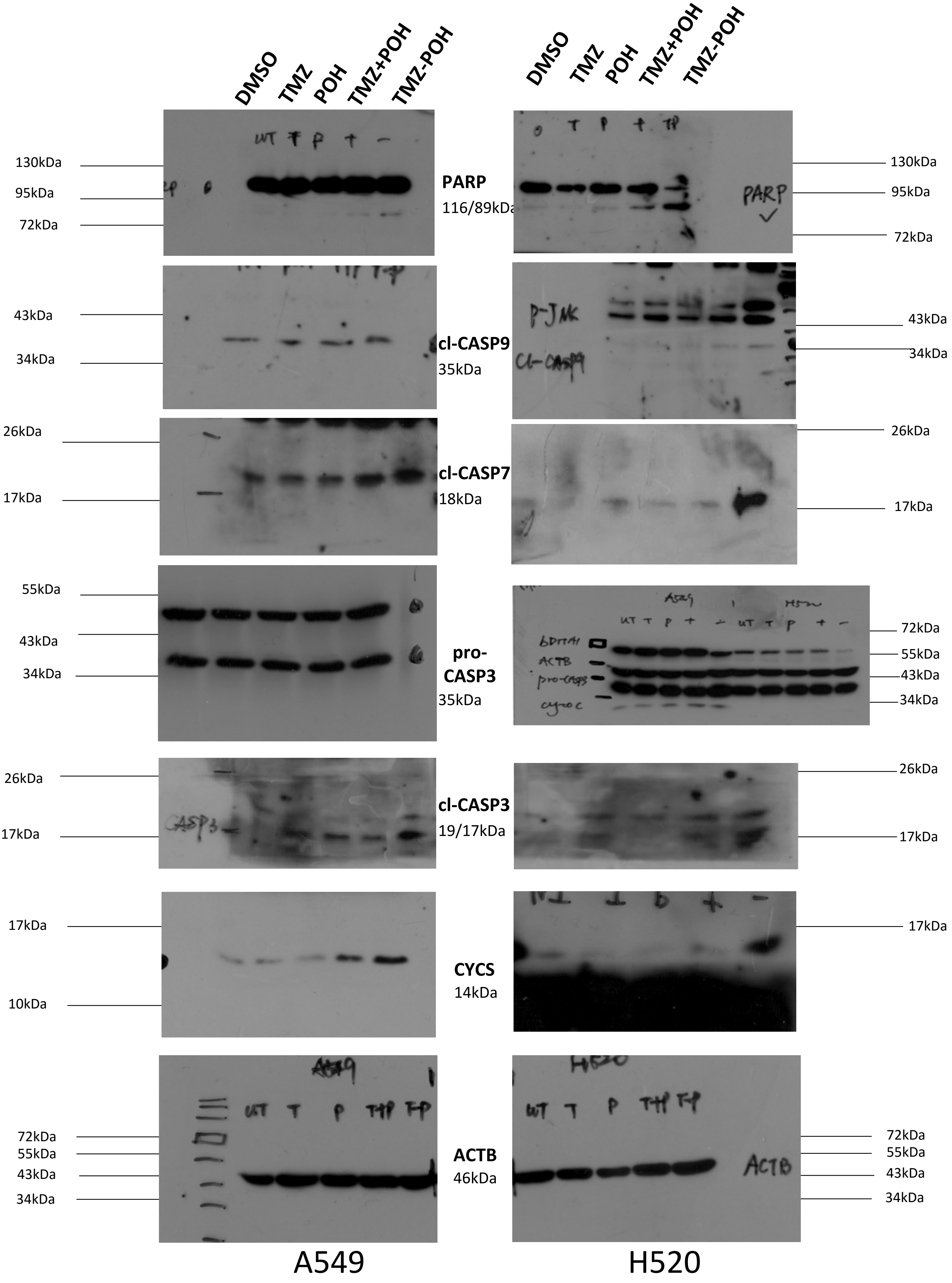

Supplement Figure S2

Supplementary File for Figure 3D

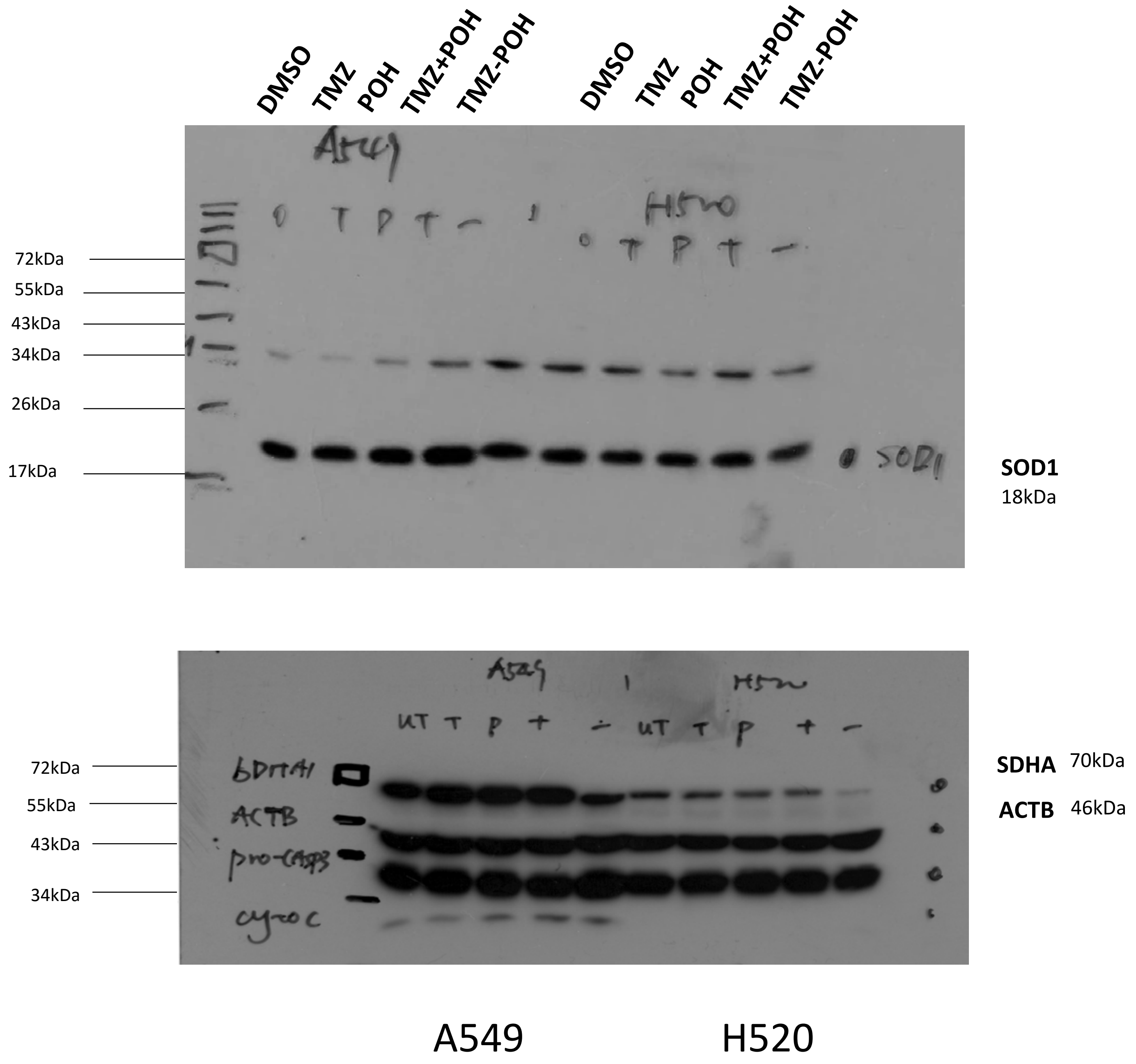

Supplement Figure S3

Supplementary File for Figure 4E

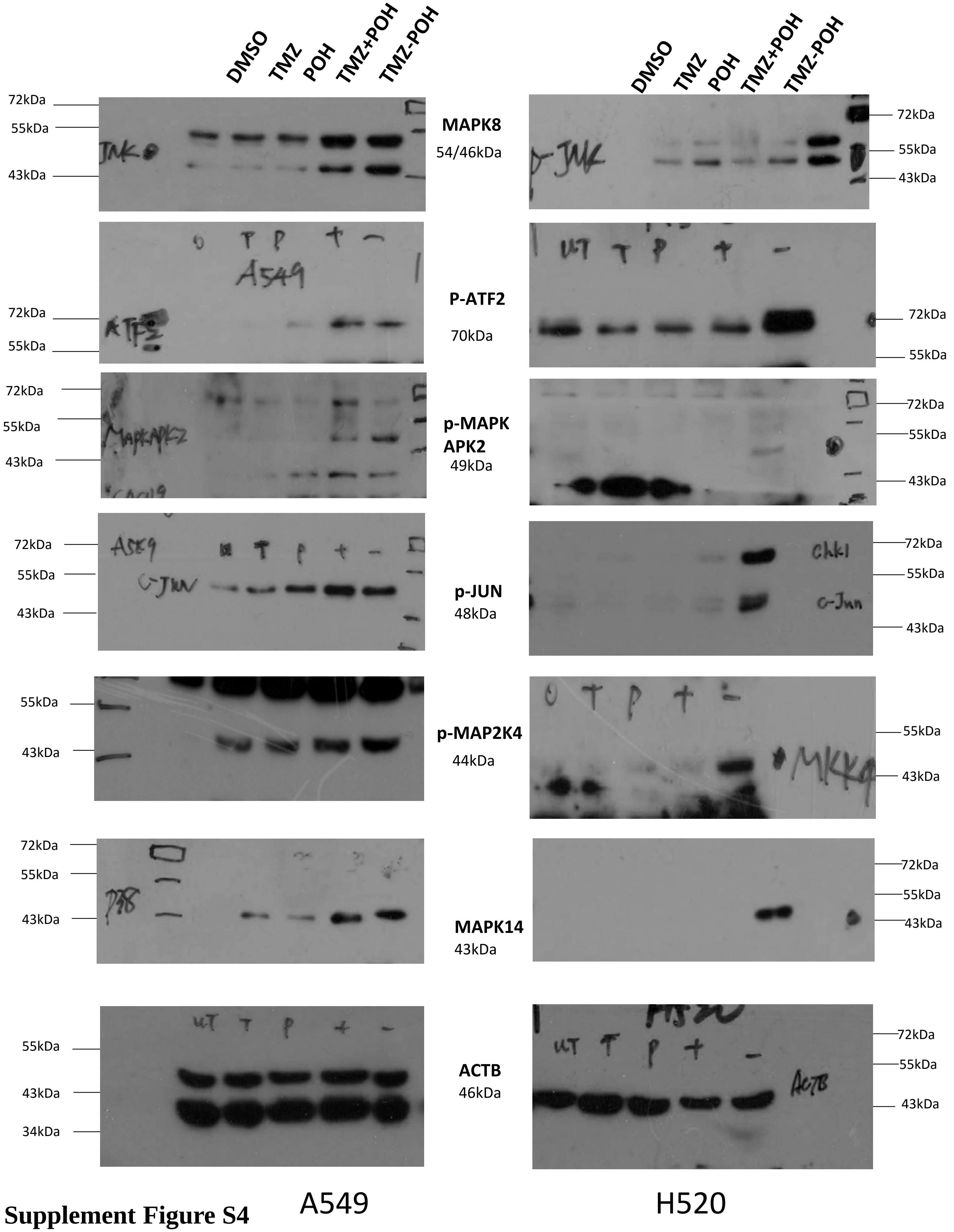

Supplement Figure S4

Supplementary File for Figure 4F

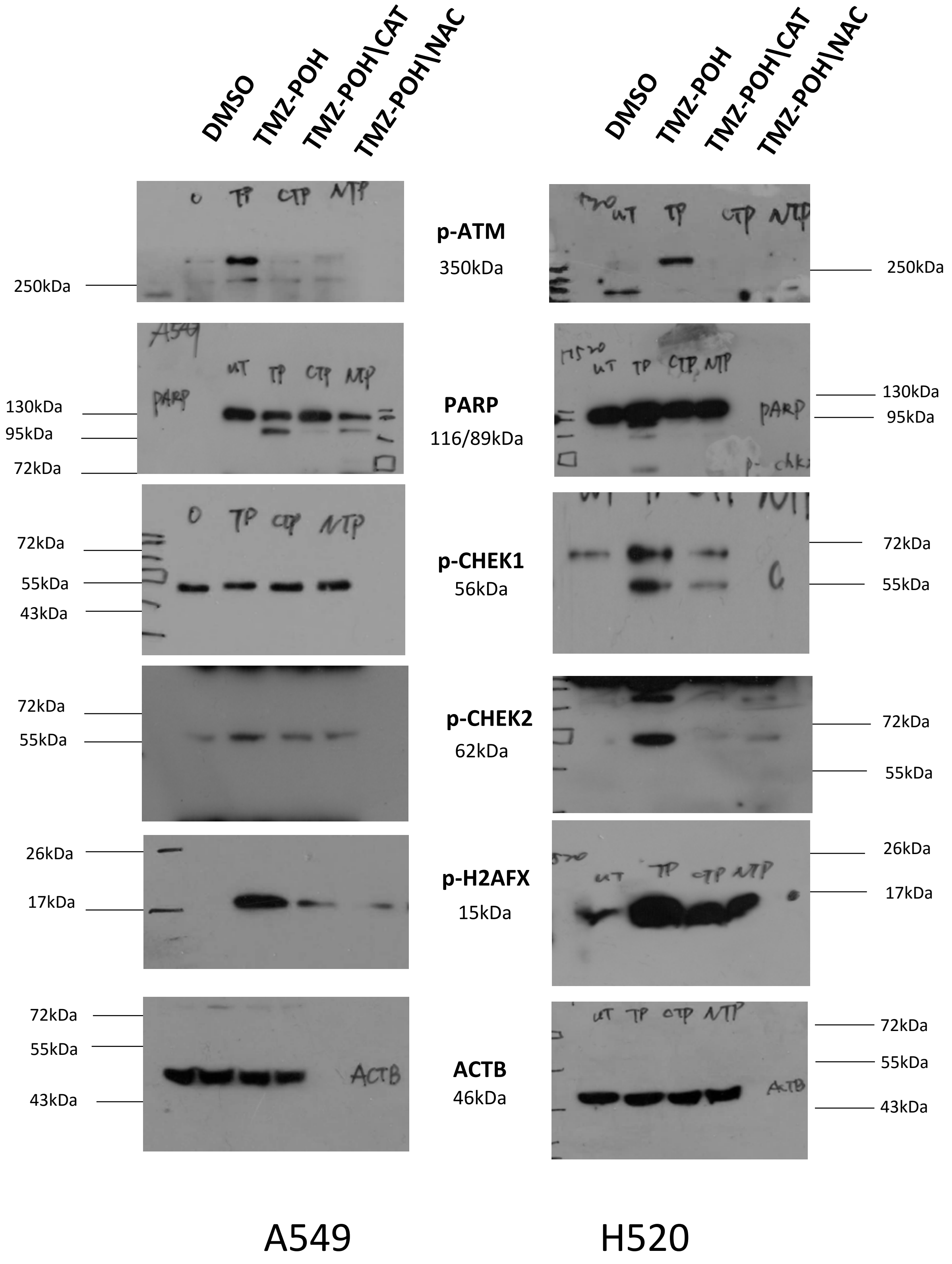

Supplement Figure S5

Supplementary File for Figure 5F
